# Magnetic resonance findings may aid in diagnosis of protracted febrile myalgia syndrome: a retrospective, multicenter study

**DOI:** 10.1186/s13023-021-02155-y

**Published:** 2022-01-10

**Authors:** Neta Aviran, Gil Amarilyo, Yaniv Lakovsky, Rotem Tal, Jenny Garkaby, Rubi Haviv, Yosef Uziel, Shiri Spielman, Hamada Mohammad Natour, Yonatan Herman, Oded Scheuerman, Yonatan Butbul Aviel, Yoel Levinsky, Liora Harel

**Affiliations:** 1 Pediatric Rheumatology Unit, Schneider Children’s Medical Center of Israel, Petach Tikva, Israel; 2 Sackler Faculty of Medicine, Tel Aviv University, Tel Aviv, Israel; 3 Department of Radiology, Schneider Children’s Medical Center of Israel, Petach Tikvah, Israel; 4 Pediatric Day Care Center, Schneider Children’s Medical Center of Israel, Petach Tikvah, Israel; 5 Department of Pediatrics B, Ruth Rappaport Children’s Hospital, Rambam Health Care Campus, Haifa, Israel; 6 The Ruth and Bruce Rappaport Faculty of Medicine, Technion - Israel Institute of Technology, Haifa, Israel; 7 Pediatric Rheumatology Unit, Meir Medical Center, Kfar Saba, Israel; 8 Rheumatology Unit, Edmond and Lily Safra Children’s Hospital, Sheba Medical Center, Tel-Hashomer, Tel Aviv, Israel; 9 Department of Pediatrics B, Schneider Children’s Medical Center of Israel, Petach Tikva, Israel; 10 Rheumatology Unit, Ruth Rappaport Children’s Hospital, Rambam Health Care Campus, Haifa, Israel; 11 Rheumatology Unit, Schneider Children’s Medical Center of Israel, 4942035 Petach Tikva, Israel

**Keywords:** Familial Mediterranean fever, Magnetic resonance imaging, Myositis, M694V, Protracted febrile myalgia syndrome

## Abstract

**Background:**

Protracted febrile myalgia syndrome (PFMS) is a rare complication of Familial Mediterranean fever (FMF). The diagnosis is based on clinical symptoms and is often challenging, especially when PFMS is the initial manifestation of FMF. The aim of this report was to describe the magnetic resonance imaging (MRI) findings in pediatric patients with PFMS.

**Results:**

There were three girls and two boys ranging in age from 6 months to 16 years, all of Mediterranean ancestry. Three had high-grade fever, and all had elevated inflammatory markers. MRI of the extremities yielded findings suggestive of myositis, which together with the clinical picture, normal CPK levels, and supporting family history of FMF, suggested the diagnosis of PFMS. Out of most common MEFV mutations tested, one patient was homozygous for M694V mutation, three were heterozygous for M694V mutation, and one was compound heterozygous for the M694V and V726A mutations.

**Conclusions:**

MRI may serve as an auxiliary diagnostic tool in PFMS.

## Introduction

Protracted febrile myalgia syndrome (PFMS) is a rare but well-described complication of familial Mediterranean fever (FMF). It is characterized by prolonged fever (4-6 weeks), elevated inflammatory markers, and severe generalized myalgia. Additional symptoms may include abdominal pain, arthritis/arthralgia, and a transient vasculitic rash [1, 2]. PFMS responds well to nonsteroidal anti-inflammatory drugs (NSAIDs) or corticosteroid treatment [3, 4]. PFMS may be the initial manifestation of FMF or it may develop in patients with known FMF, despite colchicine prophylaxis. It may appear once or recur, mimicking other complications such as polyarteritis nodosa [5].

The diagnosis is based on clinical features, with consequent genetic confirmation of a mutation/s in the *MEFV* (Mediterranean fever) gene.

The unclear etiology and pathogenesis of PFMS, as well as its dramatic presentation, often pose a diagnostic challenge, particularly in patients not known to suffer from FMF. Efforts to find other diagnostic measures have been described in isolated case reports. In 1988, Schapira et al. [6] evaluated the ultrastructural muscle tissue features of PFMS and noted a large deposition of collagen fibers during the acute stage. More recently, Fujikawa et al. [7] described a finding of fasciitis on magnetic resonance imaging (MRI) in a patient with PFMS.

Kaplan et al. [8] described a series of 15 pediatric patients with PFMS and proposed diagnostic criteria: obligatory - prior clinical and/or genetic evidence of FMF, or family history of FMF and myalgia persisting for more than 5 days; supportive - homozygosity/heterozygosity for M694V mutation, elevated inflammatory markers, and fever. These criteria do not include imaging findings. In our experience, many patients present with PFMS without known history of FMF, which complicates the diagnostic process. MRI, as a relatively available test that does not include radiation exposure and demonstrates soft tissue well, has the potential to aid in the diagnosis of PFMS and facilitate the provision of appropriate treatment. Unfortunately, there is scarce information regarding this exam in the clinical context of PFMS.

The aim of this study was to describe the MRI findings in a series of pediatric patients with PFMS who were followed at three different tertiary medical centers.

## Results

### Case 1

A 12-year-old boy of combined Egyptian-Turkish-Bukharan descent presented with 7 days of fever, prolonged excruciating abdominal pain, and pain in his right arm. Recent medical history revealed two hospitalizations in the past two years for fever, severe abdominal pain, elevated inflammatory markers, one occasion of chest pain, and arthralgia. Both episodes lasted 10 days and resolved spontaneously. Between episodes, the patient was otherwise healthy but had recurrent complaints of lower limb pain that worsened during exercise. Family history was negative for periodic fever syndromes including FMF. The patient was referred for *MEFV* genetic testing, but the parents did not comply.

Physical examination revealed fever (39.6 °C), clinical peritonitis, intense pain and swelling of the right arm without signs of arthritis, and a mild purpuric rash on the upper and lower limbs. Laboratory investigation on presentation revealed leukocytosis (WBC 21 K/microliter), markedly elevated inflammatory markers (C-reactive protein (CRP) (23 mg/dl), erythrocyte sedimentation rate (ESR) (79 mm/h), fibrinogen (840 mg/dl), von Willebrand factor 289% (normal 60-160%), and hypoalbuminemia. Muscle enzymes levels including CPK AST ALT and LDH were normal. Abdominal computed tomography-angiography (CTA) showed pleural and pericardial effusion, and a mild amount of abdominal free fluid.

MRI of the right arm (image 1.1, 1.2) demonstrated high signal intensity in T2FS in the muscles (Triceps, Biceps and Brachialis) compatible with myositis. A diagnosis of FMF with PFMS was suspected. During the investigation, NSAIDs were prescribed which led to gradual reduction of pain and resolution of the fever on day 10. He was discharged on colchicine treatment with no further episodes reported thus far. Molecular tests for FMF mutations showed compound heterozygosity for M694V and V726A.

### Case 2

An 8-year-old girl of Moroccan descent presented with 10 days of chest pain, fever, and excruciating pain in the left lower limb. Past medical history was positive for recurrent and self-resolving episodes of fever lasting for 2 days. There was no family history of autoinflammatory diseases, including FMF.

Physical examination revealed fever, and local tenderness of the left calf without any warmth, redness, or signs of arthritis. Laboratory tests demonstrated markedly elevated inflammatory markers (CRP 15 mg/dL, ESR 86 mm/h, fibrinogen 753 mg/dL) with normal CPK and other muscle enzymes. Doppler ultrasound of the left calf and isotopic bone scan were non contributory.

MRI of the calves (Images 2.1, 2.2, 2.3) showed diffuse patchy enhancement after Gd administration in the muscle, all compartments, and that were compatible with myositis. The bone marrow signal was normal. The patient’s response to corticosteroids was immediate, and she was discharged with a clinical diagnosis of FMF and PFMS. Genetic testing showed heterozygosity for the M694V mutation. Colchicine treatment was recommended.

### Case 3

A 6-month-old infant of Moroccan-Persian-Egyptian descent was referred to the emergency room with fever and restlessness of 10 days’ duration. She had positive family history of FMF. Initial laboratory results showed leukocytosis (WBC 20 K/microliter), significant thrombocytosis elevated inflammatory markers (CRP 9 mg/dL, ESR 85 mm/h), and mild hypoalbuminemia (albumin 3 g/dl); all muscle enzymes were within normal range. Extensive investigations including laboratory, metabolic, bone marrow aspirate, chest x-ray, echocardiography, abdominal sonography, and CT of the chest and abdomen were all noncontributory.

MRI of the lower limbs (Images 3.1, 3.2, 3.3, 3.4) revealed muscular atrophy. The depleted muscles show patchy high signal intensity in T2FS and corresponding enhancement after Gd injection in T1FS, findings compatible with fatty replacement and edema in the lower limbs muscles that suggest combined chronic and acute myositis. There was a spontaneous and gradual resolution of the symptoms and laboratory findings over one month, under NSAIDs treatment. Given her significant familial history of FMF, she was referred for molecular testing and found to be homozygous for M694V. Colchicine treatment was initiated. The episodic fever, restlessness, and elevated inflammatory markers together with the MRI findings suggested a diagnosis of PFMS.

### Case 4

A 9-year-old girl of Moroccan descent, previously healthy, presented with low-grade fever, refusal to walk, difficulty bearing weight on her right leg, and abdominal pain of 2 days’ duration. Family history was negative for autoinflammatory diseases.

On physical examination, the patient was afebrile, with diffuse abdominal tenderness and severe pain along the right leg, with no signs of arthritis. Laboratory results revealed leukocytosis, thrombocytosis, and elevated inflammatory markers (CRP 24 mg/dl and fibrinogen 599 mg/dl) with normal CPK. Bone scan was unrevealing and symptoms worsened to a fever of 39.5 °C and progressive myalgia in all extremities. MRI of the lower extremities demonstrated increased signal intensity of the gastrocnemius muscled, highly suggestive of myositis. Muscle biopsy was normal. Based on the combination of persistent severe myalgia, fever, elevated inflammatory markers, familial ancestry, and MRI findings, PFMS was considered. Treatment consisted of high-dose corticosteroids, with an excellent response and full clinical and laboratory recovery. The patient was started on colchicine, and later molecular testing revealed heterozygosity for the M694V mutation.

### Case 5

A 16-year-old boy of North African descent (Libya, Morocco) presented with 6 weeks of sub-febrile temperature, diffuse muscle pain, and poor appetite. Family history was positive for FMF.

Physical examination was unremarkable, other than mild tenderness of proximal muscles. Laboratory results showed elevated inflammatory markers (CRP 8.14 mg/dl, ESR 82 mm/1hr) with normal CPK.

MRI scan of the thighs showed increased diffuse signal intensity in T2FS with enhancement after gadolinium injection. These findings were suggestive of myositis. NSAID treatment was started with a rapid improvement in symptoms (<1 week) but a slower decrease in inflammation indices (> 6 weeks before normalization). A genetic test for a FMF revealed a heterozygote mutation (M964V). The diagnosis of PFMS was confirmed based on the combination of persistent myalgia, mild fever, elevated inflammatory markers, MRI findings, and genetic mutation.

## Summary of the cases

Table 1 summarizes the demographic, laboratory, radiologic, and clinical parameters of the five patients with PFMS.

**Table 1:** Demographic details, laboratory results, radiologic findings and clinical description of study patients

Characteristic	Patient 1	Patient 2	Patient 3	Patient 4	Patient 5
Age at manifestation	12 years	8 years	6 months	9 years	16 years
Sex	Male	Female	Female	Female	Male
Ethnicity – country of origin	Egypt, Turkey Bukharin	Morocco	Morocco, Persia, Egypt	Morocco	Morocco, Libya
FMF genetic MEFV mutation	Compound heterozygous M694V, V726A	Heterozygote M694V	Homozygote M694V	Heterozygote M694V	Heterozygote M694V
Total days of fever ≥ 38 DC	10	4	1	3	1
Days of hospitalization	9	12	28	23	6
Asymmetric myalgia	Yes	Yes	No	Yes	No
Corticosteroids treatment	No	Yes	No	Yes	No
CRP max value (mg/dL)	27.6	15	9.4	24.6	8.14
WBC max value (K/microliter)	19	13.3	30	29.3	11
ESR max value (mm/h)	89	81	79	ND	85
MRI findings	High T2FS signal within the arm muscles (Triceps, Biceps and Brachialis) compatible with myositis.	High signal with enhancement of the calve muscles. Normal bone marrow.	Confluent muscle edema. Muscular atrophy with fat hyperplasia.	Bilateral calf edema and increased signal intensity of the gastrocnemius muscles	Increased diffuse signal intensity in T2FS of thigh muscles with enhancement after gadolinium injection.

CRP, C-reactive protein; DC, degrees Celius; ESR, estimated sedimentation rate; FMF, familial Mediterranean fever; MEFV, Mediterranean fever gene; MRI, magnetic resonance imaging; ND, not done; PFMS, protracted febrile myalgia syndrome

Our cohort included three girls and two boys aged 6 months to 16 years. Median age at presentation was 9 years. All were of Mediterranean ancestry, and two had a family history of FMF. None of the patients were previously diagnosed with FMF. All patients had fever and significantly elevated inflammatory markers.

A comprehensive work-up was performed during hospitalization including CT and CT-angiography scans (in two cases), bone marrow aspirations, and skin and muscle biopsies. Total days of hospitalization ranged from 6 to 28 days. The diagnoses of all the patients were supported by MRI scans of the painful muscles, indicating muscle inflammation. Two of the patients were treated with corticosteroids. All patients were found to have one or two known pathogenic FMF mutations on genetic analysis performed after PFMS was diagnosed, and all were started on colchicine treatment.

## Discussion

In this study, we report on five patients who presented with the rare phenomenon of PFMS related to FMF, whose their muscle MRI findings during the acute attack were consistent with myositis. Although the final diagnosis of the patients could have been established over time without the MRI findings (combining clinical picture and genetic MEFV analysis), all described patients who presented before the diagnosis of FMF was done, and only two of them had a family history of FMF. Thus, imaging findings consistent with PFMS during the acute attack has the potential to expedite the diagnosis process and help clinician offer appropriate treatment to the painful patients.

The etiopathogenesis of PFMS is unclear. The disease is considered a manifestation of vasculitis based on skin biopsies and a dramatic response to prednisone, with substantial similarity to Henoch-Schönlein purpura [9]. Muscle appearance on biopsy, electromyography, and serum levels of muscle enzymes were all normal. Similarly, Bircan et al. [5] reported six cases of PFMS mimicking polyarteritis nodosa. Others suggested that Group A *Streptococcus* infection was a trigger of PFMS episodes [10]. A clinical association between PFMS and M694V mutation was previously described. In the study of Kaplan et al. [8], PFMS was the presenting sign in 33% of 15 patients with FMF, and the M694V mutation was found in 93% of them.

The clinical similarities to other vasculitides (fever, abdominal pain, arthritis, skin lesions) make the differential diagnosis extensive. Often costly, invasive work-up is required to exclude other diseases. Kaplan et al. [8] described a series of 15 pediatric patients with PFMS and proposed diagnostic criteria, as described above. All criteria were fulfilled in our cases.

The pathological and radiological findings in PFMS were previously described in only two case reports. Schapira et al. [6] investigated the ultrastructural picture of muscle tissue in a patient with severe myalgia. They noted infiltration of inflammatory cells and an increased amount of collagen tissue in the interstitium of the muscle.

**Fig. 1:**
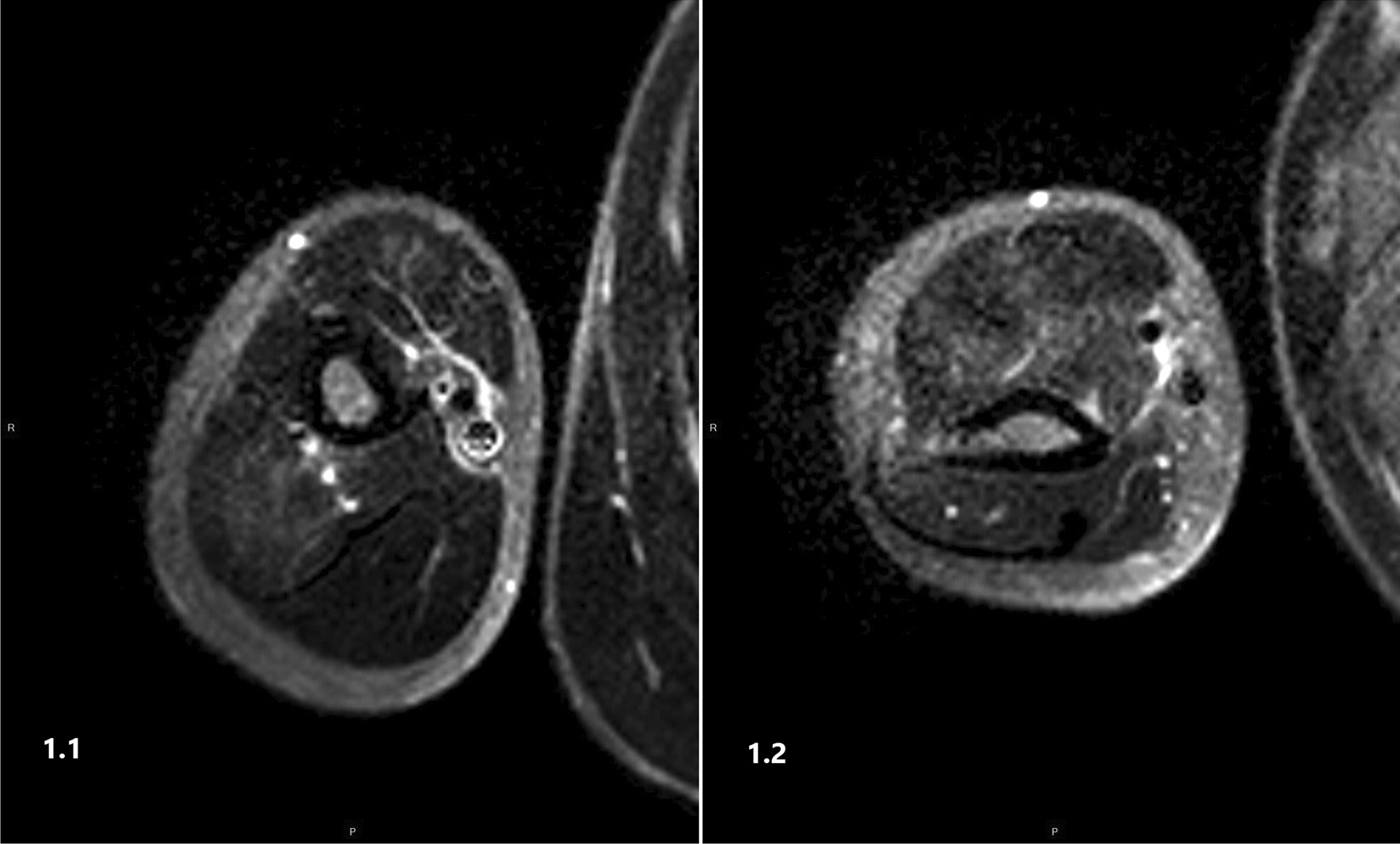
MRI of the right arm, (Images **1.1, 1.2**) of patient number 1, demonstrates a high T2FS signal (along Triceps, Biceps and Brachialis) compatible with myositis.

**Fig. 2:**
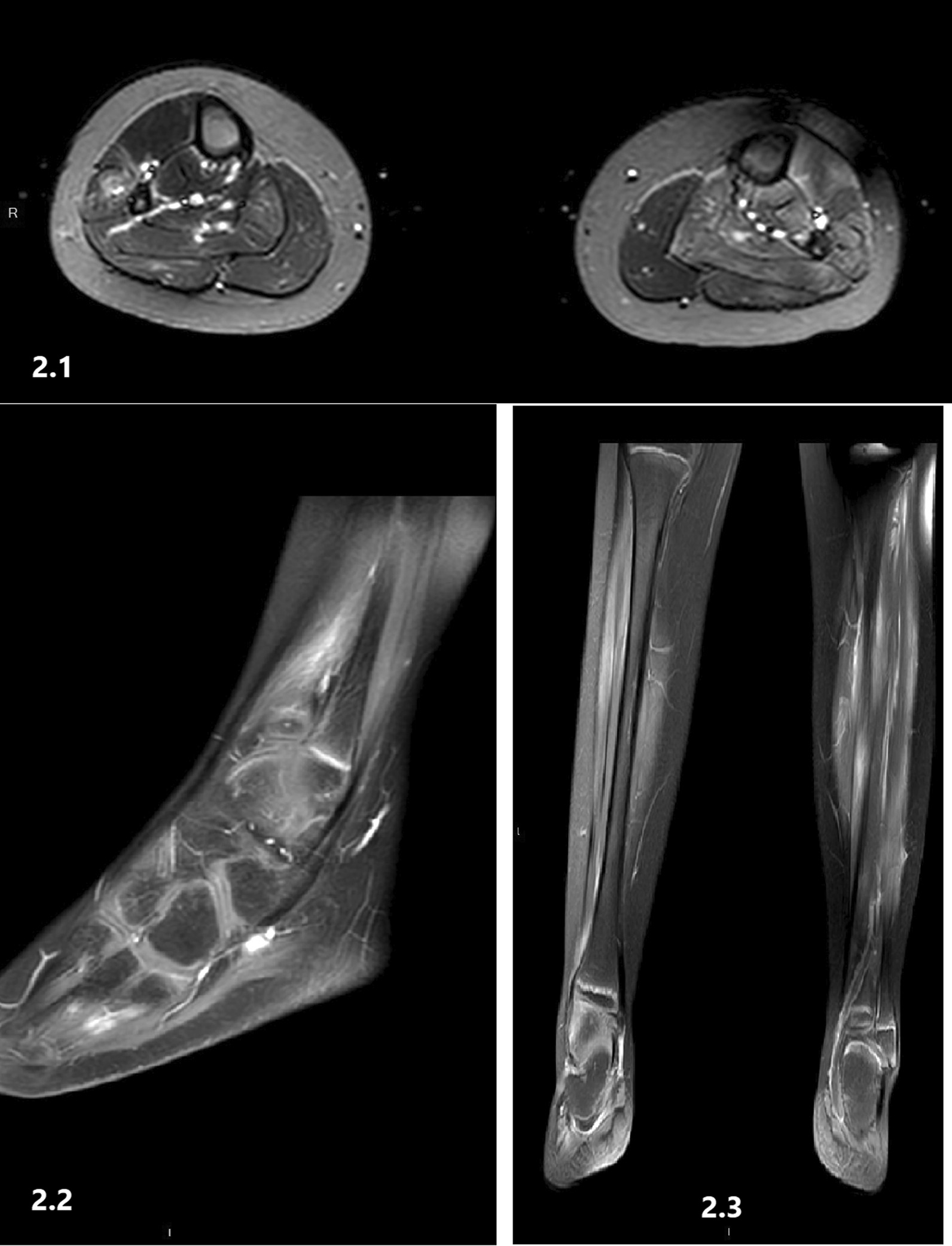
MRI of the legs (Images **2.1, 2.2, 2.3**), patient number 2, shows a high signal on T2-weighted images with calf muscle enhancement after gadolinium injection compatible with myositis. The bone marrow signal is normal

**Fig. 3:**
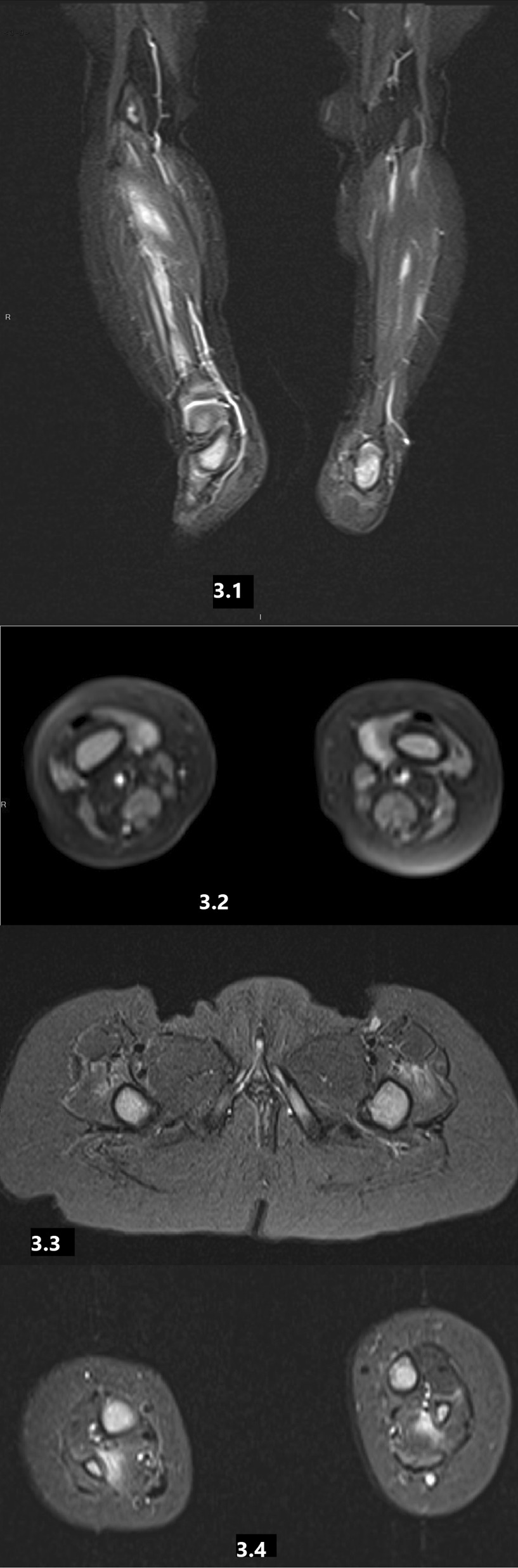
MRI of the lower limbs (Images **3.1, 3.2, 3.3, 3.4**) of patient number 3, reveals extensive edema involving the muscles without fat atrophy, compatible with myositis

A recent case report from Japan described a 22-year-old man with PFMS who showed thickening of the fascia on short-time inversion recovery (STIR) images of plain MRI [7]. The authors suggested that inflammation in the setting of PFMS occurs in the fascia rather than the muscle fiber, and that the myalgia experienced by affected patients is caused by fasciitis. All our patients showed unique findings on MRI: edema of the muscles with high intensity signal on STIR compatible with myositis. No signs of fasciitis or vasculitis were found (Figs. 1, 2 and 3).

The current study has a few limitations due to its retrospective nature and the inherent difficulty in the diagnosing this syndrome. Nevertheless, all cases met the described PFMS diagnostic criteria by Kaplan et al., and other potential diagnoses were excluded. Another limitation related to its small sample, which related to the rarity of the syndrome, even in an “endemic” FMF population as ours. Future studies are needed in order to compare the imaging findings in PFMS to findings in other muscle-involved conditions.

## Conclusion

This is the largest case series presenting MRI findings in PFMS. In many cases, PFMS is the presenting symptom of FMF. Patients undergo an invasive, prolonged workup to exclude other causes of sever myalgia accompanied by fever and elevated inflammatory markers. MRI may serve as a supportive tool for the diagnosis of PFMS, eliminating the need for extensive workup and shortening the time to diagnosis and treatment.

## Methods

A search of the database of three tertiary medical centers yielded five patients diagnosed with PFMS during 2017-2010. Data on clinical presentation and laboratory tests, genetic analysis, and MRI were collected from the medical files. In all cases, the diagnosis was based on previously proposed criteria of severe disabling myalgia of at least 5 days’ duration in a young patient with fever, elevated levels of inflammatory markers, and a positive mutation/s in the MEFV gene [8].

All cases were re-evaluated by an experienced pediatric rheumatologist [LH], to confirm the original diagnosis. MRI scans were revised by a pediatric imaging specialist with experience in musculoskeletal imaging [YL]. Key images from the imaging tests were selected for display in this article. Information about the genetic diagnosis were re-examined in the original reports in order to confirm the diagnosis in the medical records.

The study was approved by the Research Ethics Board of Rabin Medical Center.

## Data Availability

The data that support the findings of this study are available from the corresponding author, [NA], upon reasonable request.

## Abbreviations

CPK: Creatine Phophsokinase
CRP: C-reactive protein
CTA: Computed tomography-angiography
ESR: Erythrocyte sedimentation rate
FMF: Familial Mediterranean fever
MEFV: Mediterranean fever gene
MRI: Magnetic resonance imaging
NSAIDs: Nonsteroidal anti-inflammatory drugs
PFMS: Protracted febrile myalgia syndrome
T2FS: Fat-saturated T2
WBC: White blood cells
